# Coconut water vinegar ameliorates recovery of acetaminophen induced liver damage in mice

**DOI:** 10.1186/s12906-018-2199-4

**Published:** 2018-06-25

**Authors:** Nurul Elyani Mohamad, Swee Keong Yeap, Boon-Kee Beh, Huynh Ky, Kian Lam Lim, Wan Yong Ho, Shaiful Adzni Sharifuddin, Kamariah Long, Noorjahan Banu Alitheen

**Affiliations:** 1 Department of Cell and Molecular Biology, Faculty of Biotechnology and Biomolecular Science, Universiti Putra Malaysia, 43400 Serdang, Selangor Malaysia; 2 China-ASEAN College of Marine Sciences, Xiamen University Malaysia, Jalan Sunsuria, Bandar Sunsuria, 43900 Sepang, Selangor Malaysia; 3 Institute of Bioscience, Universiti Putra Malaysia, Serdang, Selangor Malaysia; 4 Biotechnology Research Centre, Malaysian Agricultural Research and Development Institute (MARDI), 43400 Serdang, Selangor Malaysia; 5 Department of Genetics and Plant Breeding, College of Agriculture and Applied Biology, Cantho University, 3/2 Street, CanTho City, Vietnam; 6 Faculty of Medicine and Health Sciences, Universiti Tunku Abdul Rahman, Sungai Long Campus, Jalan Sungai Long, Bandar Sungai Long, Cheras, 43000 Kajang, Selangor Malaysia; 7 School of Biomedical Sciences, the University of Nottingham Malaysia Campus, Jalan Broga, 43500 Semenyih, Selangor Malaysia

**Keywords:** *Cocos nucifera*, Paracetamol, Phenolic, Acetification, Inflammation

## Abstract

**Background:**

Coconut water has been commonly consumed as a beverage for its multiple health benefits while vinegar has been used as common seasoning and a traditional Chinese medicine. The present study investigates the potential of coconut water vinegar in promoting recovery on acetaminophen induced liver damage.

**Methods:**

Mice were injected with 250 mg/kg body weight acetaminophen for 7 days and were treated with distilled water (untreated), Silybin (positive control) and coconut water vinegar (0.08 mL/kg and 2 mL/kg body weight). Level of oxidation stress and inflammation among treated and untreated mice were compared.

**Results:**

Untreated mice oral administrated with acetaminophen were observed with elevation of serum liver profiles, liver histological changes, high level of cytochrome P450 2E1, reduced level of liver antioxidant and increased level of inflammatory related markers indicating liver damage. On the other hand, acetaminophen challenged mice treated with 14 days of coconut water vinegar were recorded with reduction of serum liver profiles, improved liver histology, restored liver antioxidant, reduction of liver inflammation and decreased level of liver cytochrome P450 2E1 in dosage dependent level.

**Conclusion:**

Coconut water vinegar has helped to attenuate acetaminophen-induced liver damage by restoring antioxidant activity and suppression of inflammation.

## Background

Acetaminophen or more commonly known as paracetamol is among the most commonly used mild analgesic drug worldwide [1]. Although acetaminophen is generally considered safe, unintentional or deliberate overdoses have resulted in acute liver failure especially in United States [2]. Acetaminophen is metabolized by cytochrome P450 2E1 (CYP2E1) in the liver into the reactive metabolite N-acetyl-p-benzoquinone imine (NAPQI). When consumed in safe dosage, NAPQI can be easily detoxified by glutathione (GSH) into acetaminophen-glutathione conjugate. When acetaminophen was consumed in overdose, metabolism by CYP2E1enzyme produced excessive NAPQI that depletes the liver GSH and caused mitochondrial oxidative stress. Subsequently, mitochondrial oxidative stress promotes hepatocyte cell death and release of hepatocyte contents such as ALT. Massive release of liver enzymes such as ALT also results in formation of pro-inflammatory mediators and chronic inflammation [3].

Bioactive food ingredients and herbal medicine have been widely used to alleviate chronic liver disease. The hepatoprotective effect from these food ingredients and herbal medicine was contributed by the active metabolites such as curcumin from turmeric and silymarin from milk thistle seeds [4]. Although these food or herbal ingredients have been widely consumed and generally believed as safe, their efficacy and safety will still need further validation [4]. Coconut (*Cocos nucifera* L.) water is a delicious and refreshing drink in coconut producing countries. In addition, coconut water is also consumed for various health benefits. Previous study has reported that coconut water is rich in antioxidant and lack of anti-nutritional factors. Antioxidants in the coconut water have contributed to prevent lipid peroxidation in the animal that fed with fish oil diet [5]. In addition, normal animals fed with coconut water were recorded with reduction of liver enzymes and thus proposed as potential hepatoprotective agent [6]. Moreover, coconut water has also been reported with hepatoprotection activity on carbon tetrachloride (CCl_4_) induced liver damaged [7–9] and alloxan induced diabetic [10] rat models by restoring the liver antioxidant level. However, in the coconut industry, coconut water particularly from the mature coconut was commonly handled as waste by-product due to the cost and processing flow [11]. As coconut water contained substantial amount of sugar [12], thus it is suitable as starting material to be converted as fermented end product such as vinegar.

Vinegar is widely used as food seasoning and traditional Chinese medicine [13]. It has been reported with various bioactivities including anti-diabetic, anti-hypertension, anti-microbe, liver protection and anti-tumor effects [14]. However, vinegar produced using different source of carbohydrate and strains of microbes may possess different level of bioactivities particularly on the antioxidant activity [15]. Previously, nipa water vinegar and roselle vinegar were reported with higher antioxidant level than the unfermented fruit [16, 17]. As coconut water was reported to exhibit hepatoprotective effect on CCl4 induced liver inflammation, the fermentation of this fruit water may enhance the effect. Our recent finding on hepatoprotective effect of nipa vinegar also has demonstrated a significant restoration of liver inflammation in mice treated with nipa vinegar sample [16]. Nonetheless, acetification of some fruits was reported with reduction of antioxidant activity due to decrease of total phenolics [18]. Thus, although coconut water has been reported as potential hepatoprotective agent [7–9] and coconut water vinegar has been commonly consumed to treat various disease including liver disorders and inflammation in coconut producing countries, the hepato-recovery effect of coconut water vinegar was still unclear. Thus, this study was performed to evaluate the effect of coconut water vinegar in promoting recovery of acetaminophen induced liver damage.

## Methods

### Organic acid and antioxidant level of coconut water vinegar

Coconut water vinegar (batch no:2, 9th May 2014) was obtained from Malaysian Agricultural Research and Development Institute (MARDI) (Selangor, Malaysia) in year 2013. Details of preparation method were described in Beh et al. [16]. The coconut water vinegar was standardized to 5% acetic acid and confirmed by reversed phase chromatography with eternal calibration graph (Result not shown). Organic acids in the sample were separated on an Extrasil ODS column (250 mm × 4.6 mm, 5 μm) and the detector was set at λ = 210 nm and λ = 245 nm. Determination of acetic acid was carried out at isocratic conditions at 45 °C, using a mobile phase of 50 mM phosphate solution (6.8 g potassium dihydrogen phosphate in 900 ml water, pH 2.8). The flow rate of the mobile phase was set at 0.7 ml/min. Total phenolic content and total antioxidant capacity were profiled using Folin-Ciocalteu and FRAP assays [14].

### Animals

The study was performed according to international rules and approved by Universiti Putra Malaysia Animal Care and Use Committee (IACUC) (UPM/FPV/PS/3.2.1.551/AUP-R168). In brief, a total of 35 BALB/c mice (male, 5–6 weeks old) were purchased from Animal House of the Faculty of Veterinary Sciences, Universiti Putra Malaysia and were placed in plastic cages at 22 ± 1 °C with 12 h of dark/light cycle and relative humidity approximately 60%.. The mice were maintained on a basal diet (22% crude protein, 5% crude fiber, 3% fat, 13% moisture, 8% ash, 0.85–1.2% calcium, 0.6–1% phosphorus and 49% nitrogen free extract) (Mouse pellet 702-P from Gold Coin Co, Limited, Malaysia) and were given distilled water *ad libitum*. The mice were divided into 5 groups and all mice were pre-treated with acetaminophen (250 mg/kg BW) for 7 days via stomach gavage to induce liver inflammation except for normal control group (N). The post-treatment with coconut vinegar begin after 7 days of acetaminophen induction where distilled water was given to the untreated group (UT), 50 mg/kg BW silybin was given to positive control group (S), 0.08 mL/kg BW coconut vinegar was given to low concentration treatment group (CL) and 2 mL/kg BW was given to high concentration treatment group (CH). All samples were prepared freshly prior to usage. At the end of the treatment, the mice were euthanized under ketamine-xylazine anesthesia (100 mg ketamine and 10 mg xylazine per kg body weight). Blood samples were collected from their hearts by cardiac puncture and liver was harvested for further analysis.

### Serum biochemical analysis

Serum samples were analyzed for liver marker (ALT, AST and ALP) and lipid profile (cholesterol, triglyceride, LDL and HDL) using ELISA assay kits (Roche, Germany).

### Liver tissue histological analysis

Histology of liver tissues was performed as reported previously [14]. The liver was rinsed with PBS and fixed in buffered formalin for 24 h. Then, the liver was embedded in paraffin, sectioned, deparaffinized and rehydrated using the standard techniques before further stained with hematoxylin and eosin. The morphology of the liver was then observed using bright field optic under a Nikon Eclipse 90imicroscope (New York, USA) at 40 times magnification.

### Liver antioxidant level

Excised liver was weighted and meshed in phosphate buffer saline (PBS) at a ratio of 1 g of liver to 10 mL of PBS. The supernatant was centrifuged at 10000 rpm for 10 min and the upper clear part of the supernatant was collected and kept in -20 °C prior to the antioxidant analyses. The antioxidant activity in this study was evaluated through superoxide dismutase (SOD) assay, lipid peroxidation (MDA) and glutathione reductase activity (GSH). MDA and SOD was done according to the previous study [14] while GSH was done using Glutathione assay kit according to the manufacturer’s protocol (Sigma, USA).

### Liver cytochrome P450 2E1 level

Cytochrome P450 (Abcam, USA) protein expression level was determined using Western blot technique as reported previously [14]. In brief, fresh liver tissue was weighed and meshed in liquid nitrogen before lysed in Radio Immuno Precipitation Assay (RIPA) buffer (150 mM sodium chloride, 1.0% NP-40 or Triton X-100 0.5% sodium deoxycholate, 0.1% SDS (sodium dodecyl sulphate) and 50 mM Tris, pH 8.0) added with protease inhibitor cocktail (Pierce, Thermo Fisher Scientific, USA). Protein was measured using the standard Bradford protein assay with Bradford reagent (Bio-Rad, USA). Using SDS page, an equal amount of protein was separated and transferred to nitrocellulose membrane (PALL, USA). Then, the membrane was then blocked with 5% non-fat milk (Biobasic, USA) overnight. The next day, the membrane was washed with TBST (10 mM Tris, 140 mM NaCl, 0.1% Tween-20, pH 7.6) and further incubated in primary antibody for 1 h at 4 °C followed by washing with TBST before incubated with appropriate 2° antibody for another 1 h. Then, it was washed again and incubated with HRP substrate for 10 min before viewed using a Chemidoc imager (UVP, USA). The density results obtained were analyzed using Vision Work LS Analysis software, UVP, USA.

### Liver iNOS and NF-kB mRNA expression analysis

Total RNA from liver tissue was isolated using the RNeasy kit (Qiagen, Germany). Then, first-strand cDNA was synthesized using 1 μg of total RNA in a 20 μL reverse transcriptase reaction mixture using Bio-rad iScript cDNA synthesis kit following the manufacturer’s protocol. PCR amplification was performed in a 96-well plate with a 20 μL reaction mixture containing cDNA template and 1 μM of forward and reverse primers. Quantitative real-time PCR assays iNOS and NF-kB were carried out using iQ5 (Bio-Rad, USA). The differences in CT values and the relative fold change in gene expression between groups of control and treated groups was analyzed using Bio-Rad software.

### Liver nitric oxide level

The NO activity was determined using Griess reagent kit protocol given by the manufacturer (Invitrogen, USA). Hundred and fifty μL of liver homogenate was mixed with 20 μL of Griess Reagent and 130 μL of distilled water in a 96-well plate and incubated for 30 min at room temperature. The absorbance was read at 540 nm using an ELISA Reader (Bio-tek Instrument, USA).

### Statistical analysis

Data are reported as mean ± SD and were analyzed using SPSS 16 software by one-way analysis of variance (ANOVA). K-S (with Lilliefors correction) tests in SPSS was used to test for the normality of the results in this study. Normal distribution was obtained for results in all subgroups in all tested assays. Duncan’s multiple range tests was performed as post-hoc analysis. *P* values less than 0.05 were considered significant.

## Results

### Total antioxidant and organic acids content

Comparing between fresh coconut water (total phenolic acid 167.24 ± 0.35 μg GAE/ml; FRAP: 222.87 ± 1.11 μg TE/ml) with coconut water vinegar (total phenolic acid 106.45 ± 0.01 μg GAE/ml; FRAP: 176.65 ± 0.01 μg TE/ml), total phenolic content (TPC) was recorded with ~ 36% of reduction, which has contributed to ~ 20% reduction of Ferric reducing ability of plasma (FRAP) antioxidant content. Acetic acid was not detected in fresh coconut water but the concentration of acetic acid in coconut water vinegar was 4.95% (Result not shown).

### Serum liver enzyme and lipid levels

Acetaminophen induced a remarkable increase in serum liver enzyme ALT, ALP and AST comparing to the normal healthy mice. In addition, serum cholesterol and triglyceride level were raised while HDL/LDL ratio was reduced in the untreated acetaminophen challenged mice. Silymarin and coconut water vinegar treatment were able to significantly reduced both serum liver enzymes and lipid profiles. In addition, improvement of serum liver and lipid profiles by coconut vinegar treatment in the acetaminophen challenged mice were in dosage dependent manner (Table 1).

**Table 1:** Primer sequences of inducible nitric oxide synthase (iNOS) and nuclear factor kappa-light-chain-enhancer of activated B cells (NF-kB) used in the quantitative real time PCR (qRT-PCR) assay. Beta-actin (β-actin), hypoxanthine phosphoribosyltransferase (HPRT) and glyceraldehyde 3-phosphate dehydrogenase (GAPDH) were used as housekeeping genes for normalization of the iNOS and NF-kB gene expression

	Forward primer (5′-3′)	Reverse primer (3′-5′)
iNOS	5′-GCACCGAGATTGGAGTTC-3′	3′-GAGCACAGCCACATTGAT-5′
NF-κB	5′-CATTCTGACCTTGCCTATCT-3′	3′-CTGCTGTTCTGTCCATTCT-5′
β-actin	5′-TTCCAGCCTTCCTTCTTG-3′	3′-GGAGCCAGAGCAGTAATC-5′
GAPDH	5′-GAAGGTGGTGAAGCAGGCATC-3′	3′-GAAGGTGGAAGAGTGGGAGTT-5′
HPRT	5′-CGTGATTAGCGATGATGAAC-3′	3′-AATGTAATCCAGCAGGTCAG-5′

### Histologic analysis of liver

**Fig. 1:**
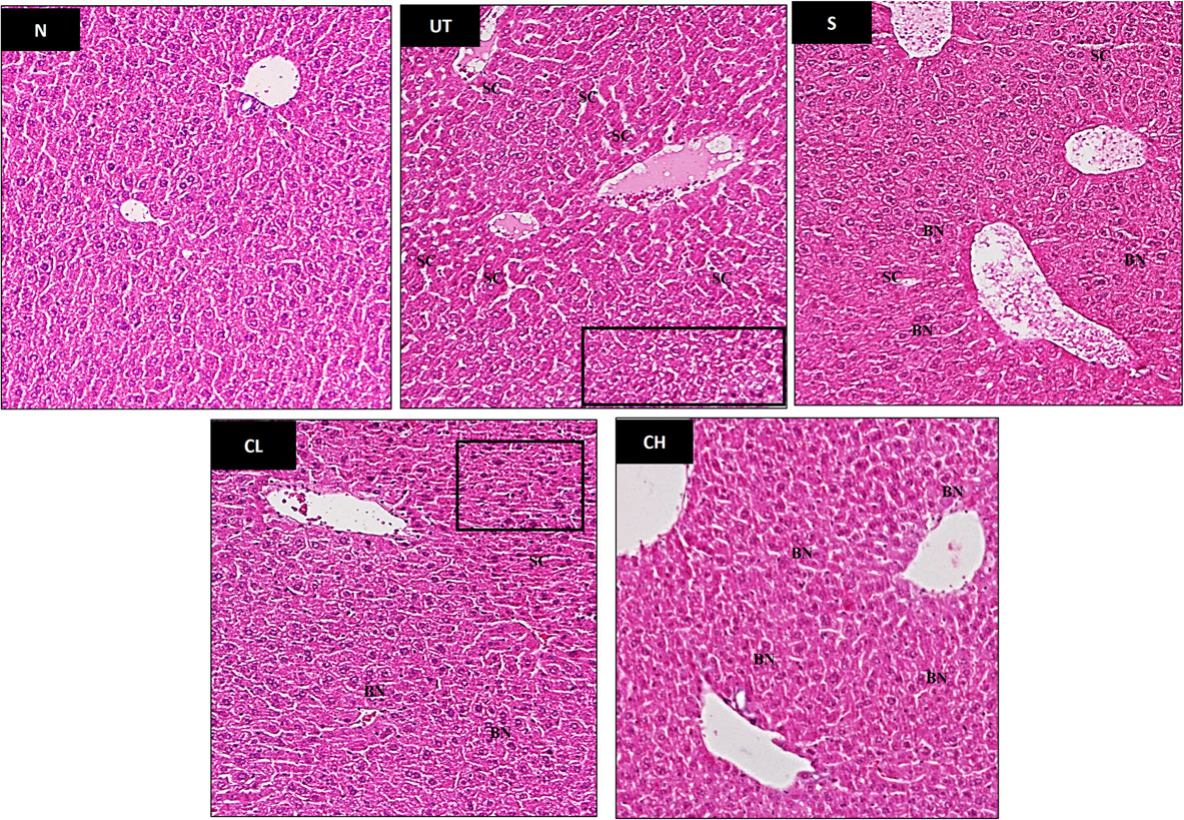
Effect of coconut water vinegar against acetaminophen-induced liver histopathological changes in mice (magnification 200×). N: Liver from normal control mice with normal histological appearance. UT: Untreated acetaminophen challenged mice with pyknotic nuclei (rectangle), and dilated sinusoidal (SC). S: Silybin treated acetaminophen challenged mice and CL: 0.08 ml/kg BW coconut vinegar treated acetaminophen challenged mice showed reduced number of dilated sinusoidal (SC) and increasing binuclear hepatocyte (BN) comparing to UT. CH: 2 ml/kg BW coconut vinegar treated acetaminophen challenged mice histological appearance similar to normal control mice with higher incidence of binuclear hepatocyte (BN)

Histologic analysis of the livers in each group is shown in Fig. 1. Dilated sinusoids (SC) and pyknotic nuclei (black rectangle) were present in the liver of untreated acetaminophen challenged mice (UT). On the other hand, mice treated with both silymarin (S) and coconut water vinegar (CL and CH) were observed with less incident of dilated sinusoids and binuclear hepatocytes (BN) indicating recovery of liver cell from the damage caused by acetaminophen. Comparing between low and high dose of coconut water vinegar, higher amount of binuclear hepatocytes and absence of pyknotic nuclei in the CH indicated that high dose of coconut water vinegar promotes better recovery.

### SOD, GSH and lipid peroxidation level in the liver

**Fig. 2:**
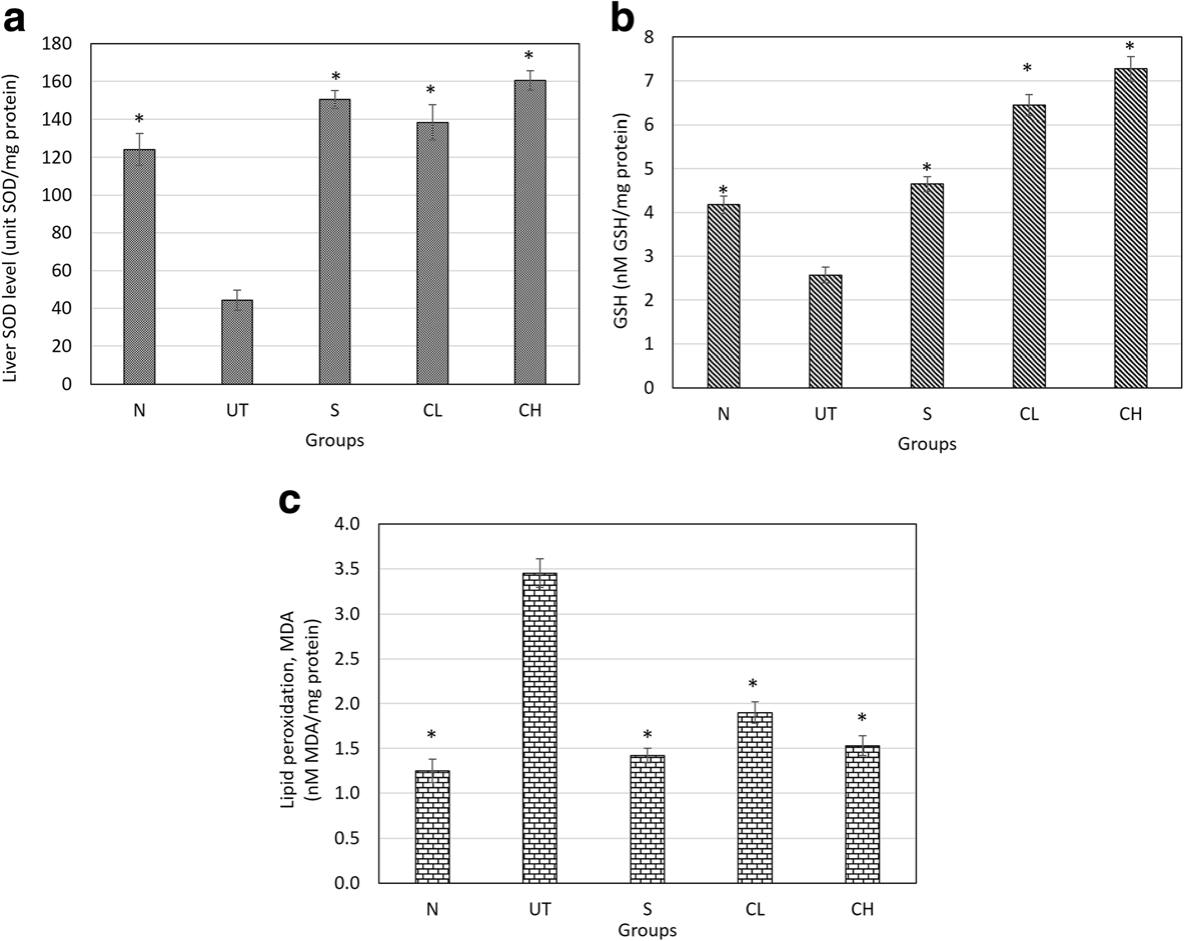
Effect of coconut water vinegar on liver **a** SOD **b** GSH and **c** MDA levels in the liver of acetaminophen-challenged mice. All values are expressed as means mean ± SD of 6 mice in each group. *P < 0.01 as compared with the untreated control group. N: normal healthy control; UT: untreated acetaminophen-induced control; S: acetaminophen-induced treated with 50 mg/kg silybin; CL: acetaminophen-induced treated with 0.08 ml/kg coconut water vinegar; CH: acetaminophen-induced treated with 2 ml/kg coconut water vinegar

Drastic reduction of SOD enzyme (Fig. 2a) and GSH peptide (Fig. 2b) level associated with increase of lipid peroxidation (Fig. 2c) was observed in the liver of untreated acetaminophen challenged mice. On the contrary, Silymarin and coconut water vinegar treatment helped to restore the SOD enzyme and GSH peptide level in the liver (Fig. 2a and b). More interestingly, GSH peptide level in both CL and CH treated mice was higher than normal healthy mice indicating that coconut water vinegar promotes antioxidant level in the mice by stimulating production of GSH in mice (Fig. 2b). Enhancement of liver antioxidant by both silymarin and coconut water vinegar were observed with significant (*p* < 0.01) reduction of lipid peroxidation in the liver as indicated by the level of malondialdehyde (MDA) (Fig. 2c).

### CYP2E1 level in the liver

**Fig. 3:**
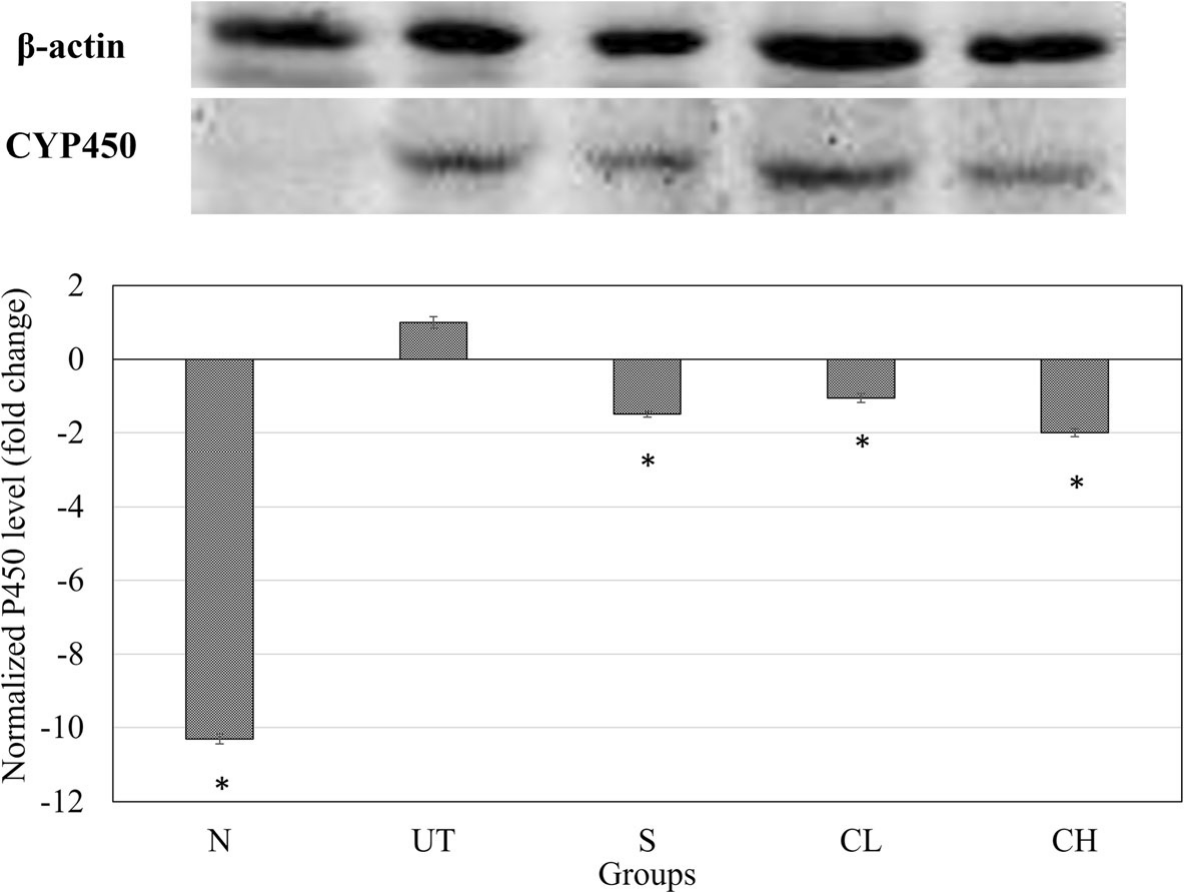
Western blot analyses of CYP2E1 and β-actin proteins in the liver. All values are expressed as means mean ± SD of 6 mice in each group. *P < 0.01 as compared with the untreated control group. N: normal healthy control; UT: untreated acetaminophen-induced control; S: acetaminophen-induced treated with 50 mg/kg silybin; CL: acetaminophen-induced treated with 0.08 ml/kg coconut water vinegar; CH: acetaminophen-induced treated with 2 ml/kg coconut water vinegar

Normal healthy mice were recorded with lowest level of cytochrome P450 2E1 (CYP2E1) level comparing to the other groups. In terms of silymarin, CL and CH treated mice, − 1.45, − 1.05 and − 2 folds down-regulation of CYP2E1 level in the liver compared to the untreated acetaminophen challenged mice, respectively (Fig. 3).

### mRNA expression of iNOS and NF-kB in the liver

**Fig. 4:**
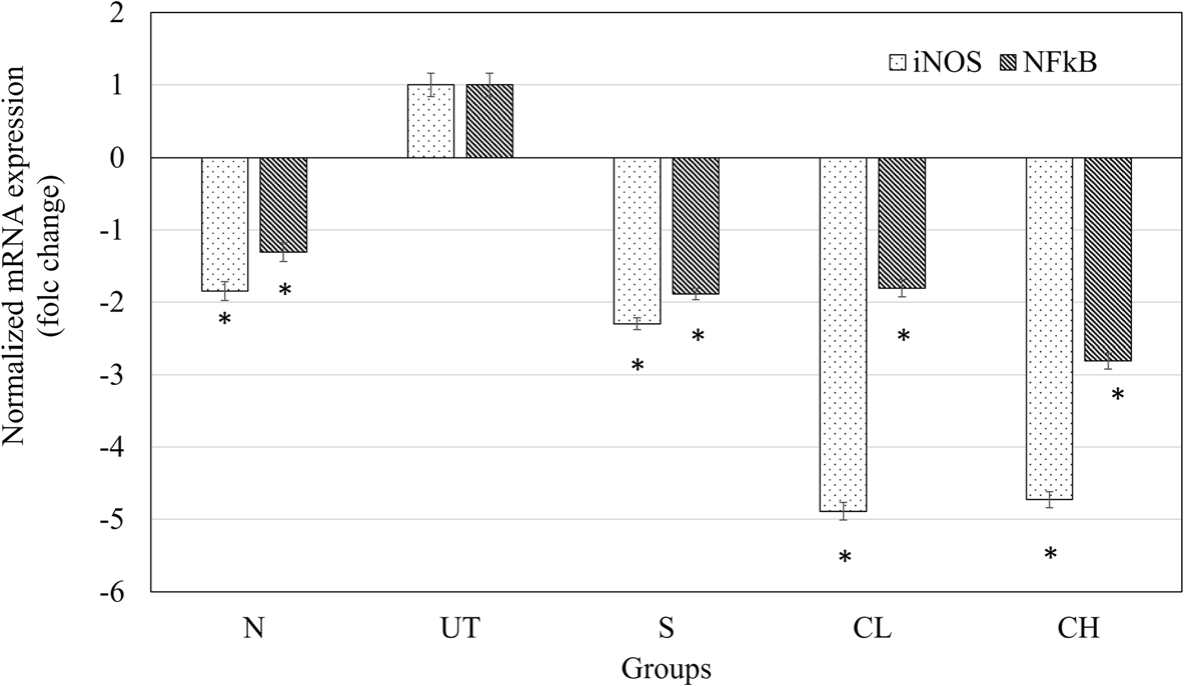
Effect of coconut water vinegar on normalized mRNA expression of iNOS and NF-kB in liver of acetaminophenchallenged mice. All values are expressed as means mean ± SD of 6 mice in each group. *P < 0.01 as compared with the untreated control group. N: normal healthy control; UT: untreated acetaminophen-induced control; S: acetaminophen-induced treated with 50 mg/kg silybin; CL: acetaminophen-induced treated with 0.08 ml/kg coconut water vinegar; CH: acetaminophen-induced treated with 2 ml/kg coconut water vinegar

The liver mRNA expression levels of iNOS and NF-kB are shown in Fig. 4. Down-regulation of iNOS and NF-kb mRNA expression were observed in the liver of normal, silymarin treated and coconut water vinegar treated mice compared to the untreated acetaminophen challenged mice (Fig. 4).

### Nitric oxide (NO) level in the liver

**Fig. 5:**
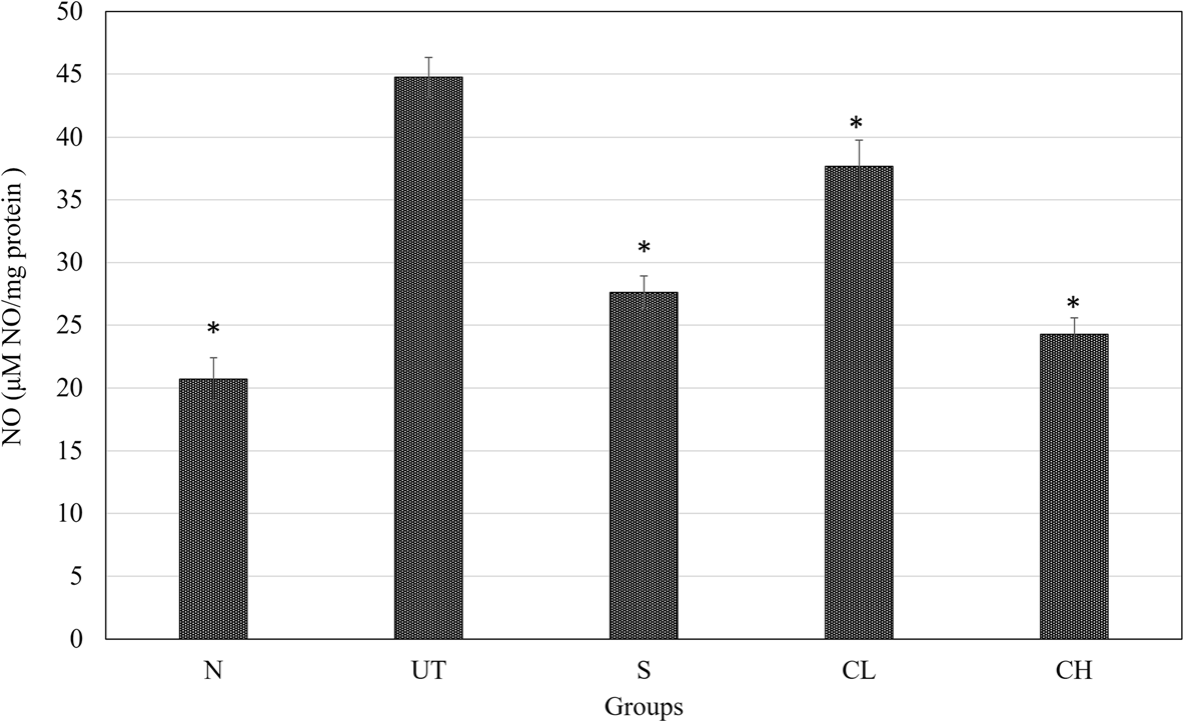
Effect of coconut water vinegar on liver nitric oxide (NO) level in the liver of acetaminophen challenged mice. All values are expressed as means mean ± SD of 6 mice in each group. *P < 0.01 as compared with the untreated control group. N: normal healthy control; UT: untreated acetaminophen-induced control; S: acetaminophen-induced treated with 50 mg/kg silybin; CL: acetaminophen-induced treated with 0.08 ml/kg coconut water vinegar; CH: acetaminophen-induced treated with 2 ml/kg coconut water vinegar

Nitric oxide (NO) level in the liver of mice untreated acetaminophen challenged mice was significantly higher compared to other groups. Silymarin and CH treatments have reduced the liver NO level close to the normal healthy mice (Fig. 5).

## Discussion

Unlike other types of vinegars [16, 17], coconut water vinegar was recorded with reduced total antioxidant activity contributed by reduction of total phenolic content as described in section 2.1. This phenomenon was common as previous study has shown that acetification of fruit that is rich in phenolic acid has slight reduction of antioxidant [18]. Previous studies have proposed that antioxidant in the coconut water contributes to the hepatoprotective effect [7–9]. Although comparatively coconut vinegar is slightly less effective than fruit vinegar, previous study has reported that even synthetic vinegar that only contained acetic acid also possessed substantial hepatoprotective and anti-inflammatory effects [14]. Thus, it is important to evaluate the hepatoprotective, antioxidant and anti-inflammatory effect of coconut water vinegar in the acetaminophen challenged mice.

In this study, acetaminophen treatment was found to cause liver damage as indicated by the high level of serum liver enzymes profile and liver histology changes [1]. In addition, elevation of serum lipid profile as shown in Table 2 also indirectly indicates mild liver function failure as liver is the major organ of fat metabolism [19]. Coconut water vinegar treatment was able to reduce serum liver enzymes level and serum lipid profiles (Table 2) indicating that liver damage caused by acetaminophen was improving after 2 weeks of treatment with coconut water vinegar. These results were supported by the higher event of binuclear hepatocytes observed in the histological study indicating liver cells underwent regeneration [20].

**Table 2:** Serum liver and lipid profiles of normal (N), acetaminophen untreated (UT), acetaminophen 50 mg/kg BW silymarin (S) treated, acetaminophen 0.08 ml/kg BW coconut vinegar (CL) treated and acetaminophen 2 ml/kg BW coconut vinegar (CH) treated mice

Group	ALT (U/L)	ALP (U/L)	AST (U/L)	Cholesterol (mmol/L)	Triglyceride (mmol/L)	HDL/LDL
N	61.23 ± 5.57*	85.67 ± 2.32*	145.20 ± 15.15*	3.30 ± 0.36*	2.33 ± 0.64*	15.93 ± 0.21*
UT	123.94 ± 7.25	104.44 ± 2.31	368.76 ± 9.83	3.75 ± 0.23	3.44 ± 0.56	13.33 ± 0.17
S	72.44 ± 8.23*	81.75 ± 1.51*	250.46 ± 11.14*	3.10 ± 0.21*	2.11 ± 0.24*	20.46 ± 0.23*
CL	39.80 ± 3.77*	75.17 ± 2.39*	163.33 ± 15.26*	2.94 ± 0.29*	2.20 ± 0.61*	18.43 ± 0.18*
CH	38.03 ± 3.35*	73.33 ± 1.52*	119.51 ± 15.49*	2.97 ± 0.36*	1.54 ± 0.37*	19.31 ± 0.23*

The data presented were representative as mean ± SD of biological replicated of mice from the same treatment group. Significant values were calculated against untreated group (*P < 0.05)

The liver damage induced by over-dosage of acetaminophen was reported due to the development of oxidative stress in the liver [1, 3]. Cytochrome P450 2E1 (CYP2E1) is the main enzyme involved in metabolism of toxic substrates such as alcohol, acetaminophen and CCl_4_. Overexpression of CYP2E1, which is commonly associated with hepatotoxicity was correlated with the increase susceptibility of apoptosis-induced liver injury [21]. During metabolism of acetaminophen by CYP2E1 enzyme in the liver, reactive N-acetyl-p-benzo-quinone imine (NAPQI), which is toxic, was formed. NAPQI can be neutralized by glutathione peptide to form non-toxic cysteine and mercapturic acid conjugates. However, over-dosage of acetaminophen can effectively deplete the GSH peptide thus caused oxidative stress in the liver [3]. Lipid peroxidation, which is normally measured by quantifying malondialdehyde, was the consequence of oxidative stress caused by acetaminophen [22]. Up regulation of lipid peroxidation contributes directly but not solely to acetaminophen overdose liver injury [23]. In this study, coconut water vinegar treatment was found to effectively restore the GSH peptide level in the liver associated with lower level of CYP2E1 and lipid peroxidation. These results have shown that although coconut water vinegar was detected with slightly lower total antioxidant capacity and total phenolic content, the in vivo antioxidant effect was not compromised. Gallic and vanillic acids were the two major phenolic acids detected in the coconut water vinegar [24]. These phenolic acids were previously reported as hepatoprotective agents [20, 25]. They may have contributed to the hepato-recovery effect of coconut water vinegar as previous studies have proposed that phenolic acids reduced the CYP2E1 expression and prevent GSH depletion via inhibiting transportation of acetaminophen into the hepatocytes via the hepatic organic anion-transporting polypeptide [26, 27].

Based on this study, overdose of acetaminophen was observed with reduction of Superoxide dismutase (SOD) enzyme (Fig. 2a). In the absence of nitric oxide (NO), superoxide that accumulates in the liver promoted lipid peroxidation mediated toxicity. However, acetaminophen overdose was also commonly observed with NO accumulation produced by pro-inflammatory reaction indicated by overexpression of inflammatory mediators including iNOS and NF-kB [1, 3] as observed in this study. Accumulated superoxide contributed by depletion of SOD enzyme preferentially react with NO to produce toxic peroxynitrite [28], which is another main agent that contributed to the hepatocyte cell death besides lipid peroxidation [23]. Coconut water vinegar was observed with anti-inflammatory effect where it suppressed the expression of inflammatory mediators’ iNOS and NF-kB, associated with lower level of NO in the liver. Concurrently, coconut water vinegar also improved the SOD activity (Fig. 2a). Thus, coconut water vinegar may have the potential to prevent peroxynitrite mediated hepatocyte damage.

## Conclusion

In summary, we concluded that acetification of coconut water to produce vinegar has significantly reduced antioxidant capacity of the coconut water vinegar due to the reduction of total phenolic content compared to the fresh coconut water. However, this phenomenon did not compensate the hepato-recovery effect of coconut water vinegar against acetaminophen induced hepatotoxicity. Coconut water vinegar promoted the recovery of liver damage induced by acetaminophen by improving the hepatic antioxidant level and suppressing the liver inflammation in dosage dependent manner. Future studies including detailed mechanisms of the coconut water vinegar hepatoprotective effect and clinical trials shall be carried out to validate the potential of coconut vinegar as a potential food supplement to ameliorate chemical induced liver damage.

## Abbreviations

ALP: Alkaline phosphatase
ALT: Alanine transaminase
ANOVA: one-way analysis of variance
AST: Aspartate aminotransferase
BN: Binuclear hepatocytes
CCl_4_: Carbon tetrachloride
CH: Acetaminophen-induced treated with 2 ml/kg coconut water vinegar
CL: Acetaminophen-induced treated with 0.08 ml/kg coconut water vinegar
CYP2E1: Cytochrome P450 2E1
FRAP: Ferric reducing ability of plasma
GAPDH: Glyceraldehyde 3-phosphate dehydrogenase
GSH: Glutathione
HPRT: Hypoxanthine phosphoribosyltransferase
iNOS: inducible nitric oxide synthase
MDA: Malondialdehyde
N: healthy normal control group
NAPQI: N-acetyl-p-benzoquinone imine
NF-kB: Nuclear factor kappa-light-chain-enhancer of activated B cells
NO: Nitric oxide
qRT-PCR: quantitative real time PCR
S: acetaminophen-induced treated with 50 mg/kg silybin
SC: Dilated sinusoids
SDS: Sodium dodecyl sulphate
SOD: Superoxide dismutase
TPC: Total phenolic content
UT: Untreated acetaminophen-induced control
β-actin: Beta-actin
